# Hemorrhagic Shock Secondary to Aortoesophageal Fistula as a Complication of Esophageal Cancer

**DOI:** 10.7759/cureus.7146

**Published:** 2020-02-29

**Authors:** Ivan Guerrero, John A Cuenca, Yenny R Cardenas, Joseph L Nates

**Affiliations:** 1 Department of Surgery, San Ignacio Hospital-Pontificia Universidad Javeriana, Bogota, COL; 2 Department of Critical Care and Respiratory Care, The University of Texas MD Anderson Cancer Center, Houston, USA; 3 Department of Critical and Intensive Care, Hospital Universitario Fundación Santa Fe De Bogotá, Bogota, COL

**Keywords:** aortoesophageal fistula, hemorrhagic shock, esophageal cancer

## Abstract

Although aortoesophageal fistulas are rare, they can present as life-threatening emergencies. This condition can develop secondary to an aneurysm, foreign bodies, infiltrating tumors, and radiotherapy. We report a patient with hemorrhagic shock secondary to an aortoesophageal fistula. A 69-year-old male with squamous cell carcinoma of the esophagus treated with chemoradiation and metallic stent placement was admitted to the intensive care unit (ICU) after an episode of hematemesis. The patient was hemodynamically unstable, requiring fluid resuscitation, blood transfusions, and respiratory and vasopressor support. The patient developed electric pulseless activity, and cardiopulmonary resuscitation was performed for 40 minutes. An upper endoscopy showed the esophageal tumor infiltrating into the stent, and computed tomography (CT) angiogram showed leakage of contrast from the thoracic aorta to the esophagus. The diagnosis of aortoesophageal fistula was made. The patient underwent endovascular management for the fistula. However, his critical condition did not improve, and the patient perished.

## Introduction

An aortoesophageal fistulae (AEF) is a rare but lethal cause of upper gastrointestinal bleeding. This condition can develop secondary to aortic aneurysms, ingestion of foreign bodies, infiltrating tumors, and surgical procedures [1]. However, recent reports have associated chemotherapy and radiotherapy with the development of AEFs [2]. The incidence of esophageal fistulas was reported to be 10%-29% in patients receiving chemoradiotherapy with esophageal cancer [2]. Due to the complexity of its diagnosis and the acuteness of the presentation, most cases are diagnosed post-mortem [1]. Typical clinical presentation, described as the Chiari triad, was defined as mid-thoracic chest pain followed by an asymptomatic interval, then a sentinel arterial hemorrhage, and, finally, a fatal hemorrhage [1]. However, the triad is not constant and requires a rigorous evaluation of the medical history, a high index of suspicion, and the addition of imaging studies to reach the final diagnosis. The need for diagnostic and management strategies is imperative. A multidisciplinary approach to mitigate the injury and offer therapeutic solutions could reduce the high mortality [3-4]. We report a patient with hemorrhagic shock secondary to an aortoesophageal fistula as a complication of esophageal cancer, radiotherapy, and esophageal stent placement.

## Case presentation

**Figure 1:**
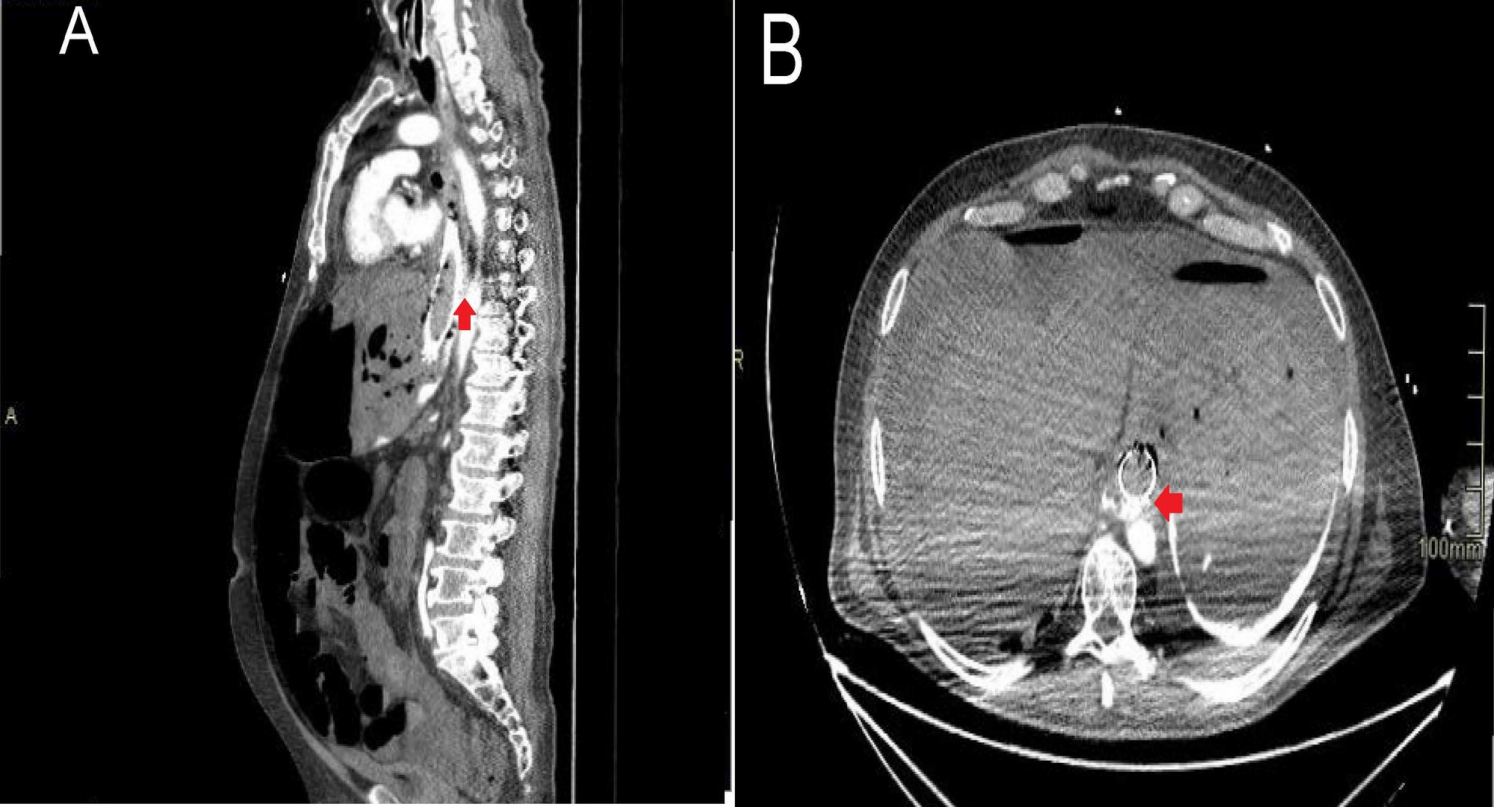
CT angiogram (A) Sagittal CT angiogram showing the leakage of contrast to the esophagus; (B) Cross-sectional CT angiogram showing contrast leak to the esophagus

A 69-year old male with stage III squamous cell carcinoma of the esophagus treated with chemo-radiation arrived at the emergency department after an episode of massive hematemesis, chest pain, and loss of consciousness. He had undergone metallic stent placement one month previous for the management of dysphagia. The patient was hemodynamically unstable with blood pressure of 74/54 mmHg, heart rate of 108 per minute, respiratory rate of 22 per minute, and temperature of 36.4°C. Hemorrhagic shock was diagnosed, and the patient was transferred to the intensive care unit (ICU) requiring fluid resuscitation, blood transfusions, and respiratory and vasopressor support. Posterior to secondary rectal bleeding, the patient developed electric pulseless activity, and cardiopulmonary resuscitation was performed for 40 minutes until the return of spontaneous circulation was achieved. Additionally, the patient exhibited abdominal distension. An emergent upper endoscopy showed the esophageal tumor infiltrating into the proximal part of the stent, and a clot adhered to the distal esophagus. Computed tomography (CT) angiogram (Figure 1) showed leakage of contrast from the thoracic aorta to the esophageal lumen. Therefore, the diagnosis of aortoesophageal fistula was made.

Treatment

**Figure 2:**
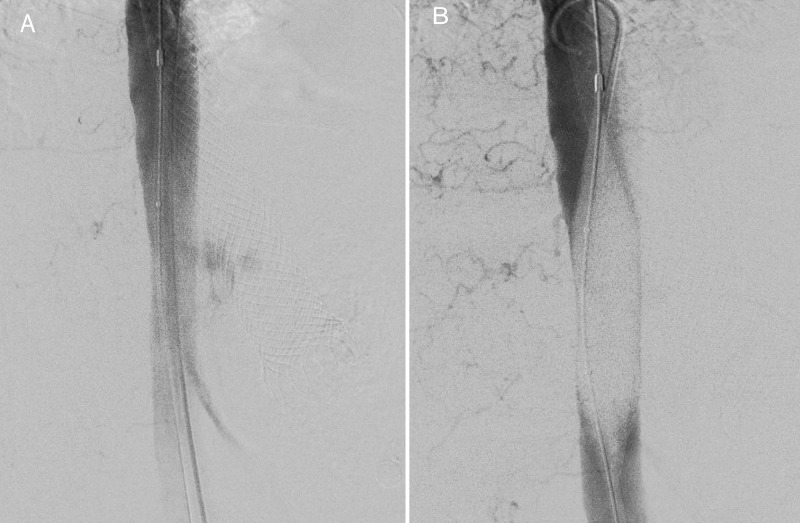
Angiography (A) Intraoperative angiography evidenced the leakage of contrast to the esophageal lumen; (B) Intraoperative angiography shows the intraluminal balloon inflated, with the stoppage of the contrast leakage towards the esophagus

After confirming the presence of the fistula, the patient underwent endovascular management, and an intravascular balloon was placed and inflated to repair the thoracic aorta. Intraoperative angiogram evidenced the correct placement of the balloon (Figure 2). Afterward, an aortic stent was placed. Despite stopping the esophageal bleeding, the patient’s critical condition did not improve, requiring norepinephrine 2.5 mcg/kg/min, vasopressin 0.1 U/min, adrenaline 1.5 mcg/kg/min, invasive mechanical ventilation, and a total of 12 units of red blood cells via transfusion.

Outcome

Due to the high requirements of life support, multi-organ dysfunction, and the irreversible nature of the baseline malignancy, a multidisciplinary conference took place. The surgical, intensive care, anesthesia, interventional radiology, and internal medicine teams discussed and decided to discontinue the escalation of care. Three hours after the patient was placed on end-of-life measures, he perished.

## Discussion

An aortoesophageal fistula is a rare entity, mainly caused by aortic aneurysms, ingestion of foreign bodies, esophageal malignancies, post-surgical complications, direct trauma, and radiotherapy [1-2]. The AEF can be classified as primary or secondary based on the etiologic mechanism. Most cases of primary AEF arise as a result of the rupture of a thoracic aneurysm. Malignancies, congenital structural anomalies, and gastroesophageal reflux are also considered primary causes. On the other hand, the secondary causes include foreign body ingestion, direct trauma, and invasive procedural instrumentation [1-2]. In our patient, multiple factors were involved in the development of the fistula. Infiltrating esophageal cancer, radiotherapy, and direct trauma associated with the previously placed esophageal metallic stent were all factors.

Exposition to radiotherapy is considered an independent risk factor for AEF. Overall, the adverse events of radiation therapy depend on several factors, including the clinical condition of the patient, the field of radiation, and doses higher than 35 Gy [2,5]. Sivaraman et al. described a case series of patients with lung cancer who developed AEF and had no evidence of residual tumor on the autopsy, leaving the exposition to radiotherapy as the cause of the fistula [6]. Although radiotherapy affects different tissues, the injury to the intimal layer is notable. While the bigger vessels are not directly compromised, the small arteries and arterioles suffer defacement of endothelial cells, fibrosis of the intima, thrombosis, and subsequent occlusion of the downstream circulation. The small capillaries supplying nutrients to the wall of major vessels can become thrombosed and result in necrosis and perforation of the subjacent main vessel, prompting the formation of the fistula [7].

Early identification and diagnosis of AEF are vital to potentially affect the outcomes since the asymptomatic period from the sentinel bleeding to the massive exsanguinating hemorrhage is unpredictable. Timely diagnosis involves a combination of imaging studies and direct observation of the bleeding. The role of emergent upper endoscopy is decisive since it is widely available. It can be performed in the emergency department, and it has high specificity and sensibility [8]. Conversely, when there is a high risk of developing massive bleeding, it is recommended to perform the endoscopy in the operating room [8]. Imaging studies with contrast such as CT angiogram and MRI can evidence the escape of contrast towards the esophageal lumen and are key to reach a definite diagnosis.

The management of AEF is directed to control the conditions involved in generating the injury to the arterial wall, prevent the continuous contamination between the fistula, and avoid infections. Conservative medical management has failed to improve survival [1]. Temporary measures, such as the insertion of the Sengstaken-Blakemore tube and embolization with isobutyl-2-cyanoacrylate, were used to stop the bleeding until surgery [9]. Endovascular techniques have shown favorable short-term outcomes [3,10-11]. Thoracic endovascular aortic repair (TEVAR), proposed by Dake et al., had a success rate of 87% and a mortality rate of 19%. However, the AEF recurrence rate and the stent infection rates were 13% and 15%, respectively [12]. The use of TEVAR improves survival as a definite measure, particularly in patients whose clinical conditions coexist with increased comorbidities and a higher risk of open surgical procedures [13]. Still, there is also evidence suggesting that the conservative approach utilizing TEVAR alone is associated with fatal outcomes due to the risk of recurrent hemorrhage and sepsis. Therefore, current recommendations support the use of TEVAR as a bridge strategy, facilitating hemodynamic stabilization before the definite surgical procedure [3,14]. Primary repair of the esophageal defect could result in positive progression, particularly in small lesions detected early [15]. Nonetheless, better outcomes are obtained with esophagectomy, removal of the devitalized tissue, in situ restoration of the aorta with a synthetic prosthesis, and protection with a viable pedicle flap of the omentum, pleura, or adventitia [9,16-17]. Depending on the cause, diversion surgery can also be necessary.

The mortality rate is still alarming, especially in cases related to esophageal cancer that require emergent thoracotomy and aortic clamping [18]. Despite the high lethality and the added risk to open intervention, there have been some cases that were successfully treated [19]. The use of extracorporeal bypass is only possible in non-hemorrhagic lesions, and it is limited to descendent thoracic aorta, leaving esophagic reconstruction for a future intervention [9].

## Conclusions

An aortoesophageal fistula is most often the result of a primary aortic aneurysm compressing the esophagus. In our patient, multiple factors were involved in the development of the fistula such as the infiltrating esophageal cancer, the trauma associated with the previously placed esophageal metallic stent, and radiotherapy. A multidisciplinary approach is needed for its diagnosis and treatment. Clinicians should consider aortoesophageal fistula in the differential diagnosis of a rapidly evolving hemorrhagic shock in a patient with thoracic cancer.
